# Italian guidelines for the prevention and management of dental trauma in children

**DOI:** 10.1186/s13052-019-0734-7

**Published:** 2019-12-04

**Authors:** Maria Grazia Cagetti, Piero Alessandro Marcoli, Mario Berengo, Piero Cascone, Livio Cordone, Patrizia Defabianis, Osvalda De Giglio, Nicola Esposito, Antonio Federici, Alberto Laino, Alessandra Majorana, Michele Nardone, Vilma Pinchi, Silvia Pizzi, Antonella Polimeni, Maria Grazia Privitera, Valentina Talarico, Stefania Zampogna

**Affiliations:** 1 Department of Biomedical, Surgical and Dental Sciences, University of Milan, Via Beldiletto 1, Milan, IT-20142 Italy; 2 Italian Society of Dental Trauma (SITD), Via Pietro Marone 16, 25121 Brescia, Italy; 3 Department of Neurosciences, University of Padova, Via Giustiniani 2, 35128 Padova, Italy; 4 Department of Oral and Maxillo Facial Science, “Sapienza” University of Rome, Via Caserta 6, 00161 Rome, Italy; 5 ASST Spedali Civili, Piazzale Spedali Civili 1, 25123 Brescia, Italy; 6 Department of Surgical Sciences, University of Turin, Via Nizza 230, 10126 Turin, Italy; 7 Department of Biomedical Sciences and Human Oncology, University of Bari, Piazza G. Cesare 11, 70124 Bari, Italy; 8 Associazione Nazionale Dentisti Italiani, Lungotevere Raffaello Sanzio, 9, 00153 Rome, Italy; 9 Unit 2, General Secretariat, Ministry of Health, Lungotevere Ripa, 1, 00153 Rome, Italy; 10 Department of Neuroscience and Reproductive and Odontostomatological Sciences, “Federico II” University, Via Giosuè Carducci, 42 Naples, Italy; 11 Department of Pediatric Dentistry, University of Brescia, P. le Spedali Civili 1, 25123 Brescia, Italy; 12 Department of Health Sciences, University of Florence, Largo Brambilla, 3, 50134 Florence, Italy; 13 Department of Medicine and Surgery, University of Parma, Via Gramsci 14, 43126 Parma, Italy; 14 Department Oral and Maxillofacial Sciences, Sapienza University of Rome, Via Caserta 6, 00161 Rome, Italy; 15 Health prevention, Italian Ministry of Health, Viale Giorgio Ribotta, 5 -, 00144 Rome, Italy; 16 Department of Pediatrics, Pugliese-Ciaccio Hospital of Catanzaro, Viale Papa Pio X, 83, 88100 Catanzaro, Italy

**Keywords:** Children, Dental trauma, First aid, Evidence-based, Guideline

## Abstract

Dental trauma is a frequent occurrence in children and adolescent and a correct diagnosis and treatment are essential for a favourable long-term prognosis. The present Guidelines aim to formulate evidence-based recommendations to assist dentists, paediatricians, surgeons, teachers, school and sport staff, parents in the prevention and first aid of dental trauma in children and to provide a careful assessment of the medico-legal implications, reviewing the first draft of the guidelines published in 2012. A multidisciplinary panel on the behalf of the Italian Ministry of Health and in collaboration with the WHO Collaborating Centre for Epidemiology and Community Dentistry of Milan, developed this document. The following four queries were postulated: 1) Which kind of precautions the health personnel, parents, sports and educational personnel must activate in order to prevent the dental trauma damage? 2) How an orofacial trauma in paediatric patients should be managed either in the Emergency Care Unit and/or in private dental office? 3) What criteria should be adopted by a dentist private practitioner to fill in a certificate in cases of dental and/or tempomandibular joint trauma occurring in children and adolescents? 4) What are the elements that should lead clinicians to suspect a non-accidental dental trauma? A systematic review and analysis of the scientific literature published in English, Italian and French from 2007 to 2017 regarding dental trauma in children and adolescents aged 0–18 years was performed, and about 100 papers were analysed and included. The following four domains were analysed and discussed: Dental Trauma Prevention Strategies and Health Education, First aid in orofacial and dental trauma, Certificate of the dental trauma, Oral and dental signs of child abuse and neglect. Twenty-eight recommendations were draw up and codified by the panel according to the Methodological handbook, produced by the Istituto Superiore di Sanità, in order to guide physicians in the prevention and first aid of dental trauma in children and adolescents. In addition, a careful assessment of the medico-legal implications is reported in this document.

## Premise

This document contains an update to the “*National guidelines for the prevention and clinical management of dental trauma in children*” published by the Ministry of Health in November 2012. An updated version of the guidelines was necessary due to the changing of the scientific evidence and the publication of research into the increasingly modalities by which dental and/or facial trauma is occurring in children, especially after animal bites. Traumas to the lower facial third that involve the temporomandibular joint (TMJ) have also been included in this document.

A lack of knowledge regarding such injuries and the consequent failure to provide adequate treatment may lead to functional deficits and aesthetic defects, especially in growing patients. As a result, articles published in the literature of the last five years on the prevention, first aid and certification of dental trauma in children have been considered.

In recent years, the prevalence of traumatic events involving the orofacial district, including the dental arcades, has increased, which has undoubtedly created a burden for public health [1]. This is due to an increasingly dynamic daily life, the increased involvement of growing individuals in play activities and competitive and non-competitive sports, the increasing use of motorized vehicles by adolescents, and the widespread habit of keeping pets, especially dogs [2].

School, the home, sports facilities and the road are where traumatic events occur with the greatest frequency. However, they are also places where it is possible to give information and put in place prevention measures. Information campaigns, through schools, sports clubs, television, newspapers, pamphlets and posters, and, above all, the internet are useful strategies for raising awareness of the prevention and first aid of dental and orofacial trauma in pre-school and school-children [3, 4].

It is also important to consider child abuse, which often involves the orofacial district; Oral and dental trauma may lead to “sentinel” event that put the dentist in the position of responding reporter [5, 6]. Likewise, special attention should be paid to self-inflicted injuries [7].

Patients who have suffered dental injury often arrive at Accident and Emergency, requiring comprehensive multidisciplinary treatment (dental, maxillofacial, paediatric, medico-legal, sports medicine, emergency medicine and preventive medicine), necessary to obtain optimal functional and aesthetic recovery. For instance, the management of trauma involving the teeth and the facial district may include the treatment of the lacerated mucosa, the reduction of maxillary fractures (possibly involving the TMJ), the immediate restoration of traumatised teeth, reattachment of fractured coronal fragments, and reimplantation of avulsed teeth, where possible. Furthermore, physicians must tailor the approach taking into account the psychological distress experienced by the patient, who is often traumatised due to the anxiety and fear provoked by the event.

Trauma diagnosis and treatment have to carry out according to specific protocols, governing a care pathway that initially involves the accurate collection of the medical history and the circumstances of the traumatic event, and continues with an initial phase of objective clinical and radiological testing, a second phase involving specific therapeutic management, and a third monitoring over time the patient.

Most of the scientific literature available today emphasises that, frequently, neither parents or caregivers nor school, sports or even healthcare personnel have sufficient knowledge of the correct procedures to be applied when a dental trauma occurs. In fact, some studies indicate that only 4% of physicians provide appropriate first aid treatment procedures [1, 8].

The prognosis of certain injuries is dependent on early and correct management. Often, the first healthcare professional to manage the dental trauma is the emergency room doctor, hospital physician or paediatrician. The role of the paediatrician is particularly important, especially in terms of guiding the proper treatment pathway, as they are generally the clinicians to whom the parents turn whenever a healthcare issue arises with their child.

## Purpose

**Fig. 1:**
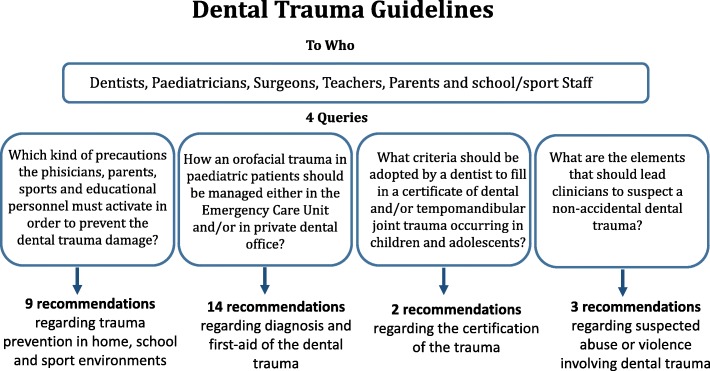
Flowcharts of Guidelines development

The purpose of this document is to provide evidence-based recommendations and indications on the prevention and first aid of dental trauma in children, and a careful assessment of the medicolegal implications. (Fig. 1).

## Fields of application

WHERE: The recommendations can be applied in the environments where a growing individual is exposed to in their daily life, and in all public and private health facilities where clinicians treat young patients who have experienced traumatic events involving the maxillofacial region.

WHO: The recommendations are aimed at GPs, paediatricians, maxillofacial surgeons, trauma surgeons, emergency room physicians, sports medicine practitioners, medico-legal specialists, dentists, dental hygienists, nursing staff, school staff, sports centre workers and parents or carers.

## Authors

This document was drawn up by a multidisciplinary panel set up for the purpose at the behest of the “Technical Group for Dentistry” (*Gruppo tecnico sull’odontoiatria*, GTO), which is part of the Italian Ministry of Health General Secretariat.

### Panel

Maria Grazia CAGETTI – University of Milan – *coordinator.*

Piero Alessandro MARCOLI – Italian Dental Traumatology Society (*Società Italiana di Traumatologia Dentale*, SITD) – *coordinator.*

Mario BERENGO – University of Padua.

Piero CASCONE “Sapienza” University, Rome.

Livio CORDONE – “ASST- Spedali Civili” local health authority, Brescia.

Patrizia DEFABIANIS – University of Turin.

Osvalda DE GIGLIO – “Aldo Moro” University, Bari.

Nicola ESPOSITO – Italian National Association of Dentists (*Associazione Nazionale Dentisti Italiani*, ANDI).

Antonio FEDERICI – Ministry of Health.

Alberto LAINO – “Federico II” University, Naples.

Alessandra MAJORANA – University of Brescia.

Michele NARDONE – Italian Ministry of Health.

Vilma PINCHI – University of Florence.

Silvia PIZZI – University of Parma.

Antonella POLIMENI – “Sapienza” University, Rome.

Maria Grazia PRIVITERA – Italian Ministry of Health.

Valentina TALARICO – “Pugliese Ciaccio” Hospital, Catanzaro.

Stefania ZAMPOGNA – Italian Society of Emergency Paediatricians (*Società Italiana di Medicina di Emergenza ed Urgenza Pediatrica*, SIMEUP).

This document was drawn up according to the guidelines provided by the Italian National Guideline Programme (*Programma Nazionale per le Linee Guida, PNLG*) [9] and underwent a process of consultation with experts from the Milan WHO Collaboration Centre for Epidemiology and Community Dentistry of Milan.

## Identification of objectives

The following objectives were identified: 1) Dental Trauma Prevention Strategies and Health Education, 2) First aid in orofacial and dental trauma, 3) Certificate of the dental trauma, 4) Oral and dental signs of child abuse and neglect.

For each of the above objectives, a series of keywords was identified and these were used alone and/or in combination.

## Literature search

A literature search was performed using the following electronic databases: The Cochrane Library, PubMed, Embase and Google Scholar.

In the first draft of the guidelines, papers published in English, Italian and French from 2007 to 2012 were included. This revised version relies on the same search criteria, extending the literature search to 2017.

Search limits: Only papers target on growing individuals (0–18 years of age) were considered for these guidelines.

## Scientific validity assessment

Full-text articles were screened using a dedicated form. The panel was divided into subgroups, each of which independently screened the articles. Any disagreements were resolved by group discussion.

## Levels of evidence

Following the scientific validity assessment, scientific evidence level tables were created, using Table 1 as a guide. This table shows the scores of the scientific evidence and describe its impact and clinical applicability, etc.

**Table 1:** LEVELS OF SCIENTIFIC EVIDENCE

I	Evidence from several randomised controlled clinical trials, systematic reviews of randomised trials and/or international guidelines
II	Evidence from one adequately designed randomised clinical trial
III	Evidence from non-randomised cohort studies with concurrent or historical controls, or meta-analyses including this type of studies
IV	Evidence from retrospective case-controlled studies, or meta-analyses including this type of studies
V	Evidence from case series with no control group
VI	Evidence based on the opinion of recognised experts or expert panels, as indicated in guidelines or consensus conferences, based on the opinion of the panel.

## Grading the recommendations

The recommendations have been codified according to Table 2 [9].

**Table 2:** RECOMMENDATION STRENGTH

A	The procedure is strongly recommended based on good-quality scientific evidence (not necessarily type I or II)
B	There are doubts about the procedure or intervention should always be recommended, but it should, nonetheless, be taken into careful consideration
C	There is substantial uncertainty regarding whether or not to recommend the procedure or the intervention
D	The procedure is not recommended
E	The procedure is strongly not recommended

## Peer-review

A list of experts in each field developed by the present guidelines was drawn up by the panel and a draft of the document was sent to all of them, asking to critically review the document and to report any error or omission. All feedbacks were discussed and amended by the panel.

## Conflict of interest

All members of the panel declare no interests conflicting with the purpose of this document.

## Dental trauma prevention strategies and health education

Query: Which kind of precautions the health personnel, parents, sports and educational personnel must activate in order to prevent the dental trauma damage?

Keywords (MeSH term): *traumatic dental injuries, public health, dental trauma, preventive strategies, mouthguards, helmet, schoolchildren, animal bite injuries, oral health promotion, oral health education, sports*

Key words have been searched individually and in association each other

Inclusion criteria: only papers answering the question above were considered. All studies based on primary and secondary prevention in children in a domesticSport and road safety context were considered for a total of 130 papers. Fifteen papers were considered relevant and added to those selected for the previous version of the guidelinesGiving a total of 30 papers included

### Introduction

Epidemiological studies show that overall, the incidence/year of dental trauma stands at 4.5%: approximately a third of children and toddlers and a quarter of adolescents and adults [10].

The prevalence of dental trauma varies from 6.1 to 62.1% in pre-school children and from 5.3 to 21% in schoolchildren [11].

Traumatic events responsible for dental trauma can be found at home (stairs, wet floors, sharp corners) [2, 12, 13], at school [14] in sports setting (collisions, elbowing and falls) [15] and during free-time activities (bicycle rides, walking in woods and on the beach, use of roller skates, skateboarding and rollerblading, etc).

Specific traumatic events to the maxillofacial region can occur in public places (slippery surfaces, road works badly-maintained roads, and as a result of animal bites, etc).

#### Prevention

Observational studies have suggested that dental trauma can cause pain, functional impairment and aesthetic problem, with physical, emotional and social consequences for children and their families. This indicates the need for primary, secondary and tertiary prevention programmes in children and young adults [4].

The implementation of suitable primary measures of prevention with the aim of protecting healthy subjects depends on correct information being managed between dentists, orthodontists, dental hygienists, parents and carers, school and sports teachers working closely with paediatricians.

Secondary prevention measures must, however, be implemented when damage has occurred and are designed to limit the harmful consequences by means of a careful clinical evaluation and a correct treatment of the dental trauma [16].

Finally, tertiary prevention, strictly related to orthodontic treatment, has the aim of reducing complications and restoring chewing, aesthetic and phonetic functions.

#### Primary prevention at home

Babies, children and elderly people are the categories of people most at risk from domestic accidents.

For individuals within 14 years of age, the living room (area of the house dedicated to free time and games) is the most at-risk environment (74.6% of domestic accidents) [17].

In early childhood, starting to walk, with or without a walker is the most frequent moment when dental trauma can occur as a result of the greater possibility of falling and hitting furniture [18].

Young and obese subjects also show a greater tendency to fall [19]; although the data currently available are not sufficient to establish a causal relationship between dental trauma, physical activity and nutrition [20].

In individuals showing an increased overjet (the distance between upper and lower incisors in the anterior-posterior direction) as occurs in subjects who habitually suck their thumbs or a dummy, dental trauma is more common in the case of “face first” falls [21–23]. It is therefore important to have specialist orthodontic examinations to identify and correct early possible dental protrusions [24].

In all these subjects and particularly in lively individuals the use of mouthguards would be appropriate [12].

Oral trauma in infants can also be caused by animal bites (mainly cats and dogs), which, in addition to the physical and psychological damage, can cause infections [25]. The detection and removal of teeth or fragments of teeth is crucial in reducing significantly the risk of infection [26, 27].

In particular, prevention strategies against dog bites should include careful supervision of the interaction between infants and dogs, education relating to the responsibility of dog owners and observance of the rules relating to the control of animals.

It is also necessary to increase knowledge in the community of the rabies virus and the necessity to vaccinate animals against it [25].

Recommendation 1

It is important to adopt measures to discourage non-nutritional sucking by two years of age in order to completely arrest the habit in the following year**,** since it is a risk factor for the overjet increase.

Recommendation strength: A.

Level of evidence: I.

Recommendation 2

In the case of increased overjet, parents should be informed of the risk of dental trauma caused by face falls and suggest an orthodontic evaluation.

Recommendation strength: A.

Level of evidence: IV.

#### Primary prevention at school

Given the frequency of dental trauma in a school environment it would be useful for educational institutions to organise training courses for teachers, school employees and students with the aim to identify and reduce risks of dental trauma in children [28, 29].

The choice of the teaching method should take into account technological developments, of which the audio-visual supports have been shown to be most effective in communicating educational messages [30].

The most common traumas are a consequence of the habit of chewing pens and keeping stationery items in the mouth, fights [19], the playing of games that use blunt objects and falls during sports and recreational activities.

In all schools a first aid area should be present together with conditions to facilitate immediate intervention in the case of dental trauma.

Recommendation 1

It is recommended that training is given to teachers and non-teaching staff regarding the risk of dental trauma. These training courses should use videos, brochures and /or manuals. Posters and leaflets explaining risks should be put on walls of gyms and swimming pools.

Recommendation strength: A.

Level of evidence: VI.

Recommendation 2

It is recommended to inform and teach children regarding the risk of dangerous behaviour and its consequences.

Recommendation strength: A.

Level of evidence: VI.

#### Primary prevention in sport ENVIROMENT

Contact sport such as basketball, volleyball, football rugby and horse-riding but also non-contact sports such as swimming (wet surfaces surrounding the swimming pool) or artistic gymnastics and other sports activities such as cycling, using skateboards, roller skates or rollerblades and skiing represent some of the activities with an elevated risk of dental trauma in young people mainly as a result of falls or collisions with other participants [31, 32].

In these cases, both dentists and sports centre staff might suggest the use of helmets, mouthguards and/or facemasks suitable for the sport being practised, informing players of the risk of dental and facial damages and of the benefits of these safety measures [16, 33–35].

#### Secondary prevention

In the case of dental trauma the patient should be kept calm, any blood should be cleaned away and the nature of the damage caused should be evaluated carefully (type of teeth involved, dental fractures, mobility or partial extrusion of teeth, possibility of salvaging any tooth fragments or the entire tooth) and the type of first aid required (need for avulsion, replacement, reattaching the fragment/s, protection of the tooth pulp and/or the exposed tooth).

An avulsed tooth has a good probability of being reinserted if it has been kept in liquid solutions (physiological solution, milk or saliva). The prompt reinsertion is the procedure that has the highest percentage of success [10]. In addition, there is a good possibility of healing if the reinsertion, temporary splinting to other teeth (for 2 to 4 weeks) and further check-ups are carried out correctly [12, 36].

In all cases, a follow-up protocol is necessary in order to avoid complications arising.

If the event has occurred in a very early age, this represents a valid reason for a dental examination and a series of follow-up appointments during the following year, with the aim of reassuring the child of completely recovering the aesthetic of the smile.

Recommendation 1

It is recommended to include sterile saline solution vials in first-aid kits.

Recommendation strength: A.

Level of evidence: III.

Recommendation 2

In cases of fracture of the crown of the tooth

1. it is recommended to retrieve the broken fragment.

2. it is recommended to go immediately to an Emergency Department with a dental service, or to a private dentist. When the tooth that has been avulsed is permanent, the reinsertion is recommended. If the avulsed tooth is a primary tooth, the reinsertion is not recommended.

Recommendation strength: A.

Level of evidence: I.

## First aid in orofacial and dental trauma

Query: How an orofacial trauma in paediatric patients should be managed either in the Emergency Care Unit and/or in private dental office?

Keywords (MeSH term): *traumatic dental injuries, dental trauma, paediatric facial trauma, TMJ fractures, children, adolescents, first-aid trauma, emergency*

Key words have been searched individually and in association each other

Inclusion criteria: only papers answering the question above were considered. A total of 53 papers were foundAnd 22 were selected. Finally 39 papers were includedSince some of the references of the previous version of the guidelines were eliminated and/or substituted by the new ones.

### Introduction

The efficacy of the management of oral-facial injuries in paediatric dentistry depends on a prompt and accurate diagnosis, followed by an appropriate treatment to improve the prognosis. In case of crown fracture or avulsion, the prognosis depends mainly on a quick and appropriate treatment to improve short and long-term outcome [37]. Signs and symptoms referred by patients must always be carefully evaluated to exclude systemic and/or local (orofacial region) complications. For this reason, it is necessary to manage all the most appropriate procedures to face the trauma since the first aid approach [37, 38].

Many papers concerning orofacial trauma in paediatric dentistry have been recently published [39, 40]; however, published papers do not fully address all the different clinical problems of dental traumatology, limiting to dealing with single aspects [41–44]. As a result, a useful and practice guidelines for clinicians (paediatricians, Emergency Care Unit staff or dentists) who face orofacial trauma in an emergency setting is needed [45].

Recommendation 1

Fragment/s of tooth/teeth and avulsed tooth/teeth should be put in a proper storage medium (milk, saline solution, saliva) before the replantation as recommended by 2011 AAPD and 2012 IADT Guidelines [36, 46–48]**.**

Recommendation strength: A.

Level of evidence: I.

Initial evaluation at the emergency care unit

The first classification of a traumatic event and its priority code are given during the first evaluation of the trauma at the Emergency Care Unit [49, 50].

1.1. Trauma History
  All information concerning the traumatic event must be collected. This includes:

- the place where the injury occurred (school, home, gym, road, etc.)
- the cause of the trauma (accidental fall, car accident, aggression, etc.)
- the dynamics of the traumatic event.
- the time and interval period existing between the event and the first aid.
-signs and symptoms referred by the patient.
- recovery of avulsed tooth/teeth or tooth/teeth fragments and the medium used for the storage.

Recommendation 2

It is recommended to perform a scrupulous medical history targeting the following: the dynamics, place and modality of the traumatic event; the symptoms referred at the moment of the trauma, such as pain, loss of consciousness, confusional state, amnesia, headache, nausea/vomit, alterations of the sight, convulsive crisis, speech difficulties, bleeding and otorrhea; if tetanus vaccine is up to date.

Recommendation strength: A.

Level of evidence: IV.

Recommendation 3

The remote pathologic anamnesis, personal and familiar, must be precise, punctual and mainly targeting the hematologic diseases (clotting blood disorders), and/or pathologies which may influence treatment options (such as congenital heart diseases requiring antibiotic prophylaxis for bacterial endocarditis) or allergies.

Recommendation strength: A.

Level of evidence: III.

### Clinical examination of the patient

In children the general physical examination, even in case of dental trauma, is very important since, in young patients, symptoms are not well described and systemic signs might be not clearly expressed [51].

Paediatricians and paediatric dentists must focus their attention on signs resulting from cervical and/or cranial trauma, such as bruises in the mastoid region, central or peripheral neurological problems, etc. [52–56]. In addition, injuries that may lead the clinician to suspect a child abuse (such as bruises which do not correspond to bone prominences, lesions due to recognizable objects, bites, burnings, injuries of different timing etc) must always be carefully considered [25].

Examination should also include the evaluation of jaw movements: mandibular range of motion (i.e. maximum unassisted opening, maximum assisted opening, maximum lateral excursion, maximum protrusive excursion) and opening pattern on the frontal plane (i.e. symmetrical vs asymmetrical) must be considered carefully. A functional examination should also include the evaluation of occlusion: the presence of occlusal alterations such as posterior pre-contacts, open bites, etc. must be recorded as they may be the result of temporo-mandibular joint (TMJ) involvement [57–59]. Underestimated TMJ fractures may result in abnormal facial growth, asymmetries and/or mandibular micrognathism [60, 61].

Teeth’s position must be always examined in order to show up injuries with or without pulp exposure, displacement and/or tooth mobility.

Extra-oral examination must always consider:

- clinical signs which may imply cranial or cervical trauma;
-lesions non-corresponding to bone prominences such as bruises, lesions due to recognizable objects, bites, burnings, injuries of different timing etc.
- presence of facial asymmetries;
- pain assessment (particularly during mandibular movements);
- evaluation of jaw movements, as reduced/deviated mouth opening may be the result of TMJ fractures, effusion and/or bleeding in the joint space.
- presence of bruises, lacerations/abrasions of perioral soft tissues.
- The intraoral examination [62–68] must always consider:
- teeth fractures (with or without pulp involvement), teeth dislocations, mobility or avulsion.
- soft tissue conditions, with special attention to oral mucosa, gingiva or tongue injuries;
-careful assessment of post-traumatic occlusal alterations such as open bite and posterior pre-contacts.

Recommendation 4

In addition to the examination of the orofacial area

- a general assessment of patient’s condition must always be considered;
- the presence of cranio-cervical injuries must be recorded;
- oral mucosae lesions and extra oral injuries pathognomonic for child abuse must always be recorded;
- the assessment of the mandibular dynamics is recommended.

Recommendation strength: A.

Level of evidence: III.

### Radiological diagnosis

In case of suspected bone fractures, face radiograms such as panoramic X-ray (OPG), lateral (LL-TRX) and postero-anterior cephalograms (AP-TRX) and reverse Towne’s views are very useful. The latter does not require a lot of child’s cooperation and it is recommended in very young patients because of the low quantity of radiation delivered. Other methods of advanced imaging may be needed [69–71].

Recommendation 5

The radiological investigation of the facial district is necessary in order to exclude or confirm the presence of bone fractures. It is highly recommended in the case of direct or indirect facial traumas involving the orofacial region or the mandible. Other imaging investigations might be needed in case of multiple traumatic lesions and/or suspected child abuse.

Recommendation strength: A.

Level of evidence: III.

### Therapy

Facial trauma requires a multidisciplinary approach: mid-face and mandibular fractures treatment is the responsibility of the maxillofacial surgeon, while dental and oral soft tissues lesions are the responsibility of the dentist. In case of trauma involving children and adolescents, only parents and/or caregivers can give consent to dental treatments [72, 73]. If needed, urgent diagnostic and therapeutic procedures (such as sutures of a wound, reduction of displaced teeth, tooth replantation, treatment of the exposed dental pulp, etc..) must be, however, done in order to prevent worse outcomes. Postponed treatments always need parents or caregivers consent.

Recommendation 6

In case of facial trauma, a specialist consultation is highly recommended to evaluate if the treatment of the traumatic lesions can be delayed or not (as in the case of tooth avulsion, pulp exposure etc.).

Recommendation strength: A.

Level of evidence: IV.

First dental assessment

The first assessment of a dental trauma in a child must provide a classification of the type of trauma in order to set up proper diagnostic and therapeutic procedures.

### History

A) If the patient has already been examined at the Emergency Care Unit
  The dentist must evaluate the clinical and radiographic examination reports given by the Urgent Care Unit
B) If the patient has not been examined at the Emergency Care Unit
  The first dental assessment includes the self-report history of the traumatic event and the clinical examination of the patient.

#### History of the traumatic event

All the information concerning the traumatic event must be recorded, with special attention to:

- where the traumatic event occurred (school, home, gym, road, etc.);
- cause of the trauma (accidental fall, car accident, aggression, etc.);
- self-reporting of the traumatic event;
- timing of the trauma and time interval between the traumatic event and first aid;
- signs and symptoms reported by the patient;
- the presence of dental fragments for possible reattachment and/or presence of avulsed tooth/teeth suitable for immediate replantation (if stored in a proper medium) must always be carefully evaluated.

Recommendation 7

Complete medical and dental history must be recorded (see Recommendation n. 1).

Recommendation 8

If the patient showed systemic symptoms when the trauma occurs or during the dental examination, he/she should be immediately sent to the Emergency Care Unit for proper evaluation.

Recommendation strength: A.

Level of evidence: VI.

Recommendation 9

The presence of previous systemic diseases must always be investigated (see Recommendation n. 3).

### Clinical examination

#### Extra-oral examination

- Patient already examined by the Urgent Care Unit staff must be evaluated for:

- facial asymmetry
- pain
- reduced / altered temporo-mandibular joint (TMJ) mobility
- reduced / altered mandibular movements
- presence of ecchymosis, lacerations or abrasions of peri-oral soft tissues

b) Patient NOT examined by the Urgent Care Unit staff must be assessed for

- Clinical signs of head or cervical trauma
- Lesions such as ecchymosis, lesions due to recognizable objects, bite marks, etc. which may result from mistreatment or abuse
- Reduced mouth opening and deviation during mandibular movements pathognomonic of TMJ involvement (fractures, dislocations, endo-articular effusion)
- Pain (particularly when exacerbated by mandibular movements)
- Facial asymmetry
- Peri-oral soft tissues lesions such as lacerations or abrasions

#### Intra-oral examination

The dental assessment of traumatized patients, no matter if examined or not by the Urgent Care Unit staff, must always include evaluation of teeth and periodontal tissues conditions with special attention to:

- primary / mixed/ permanent dentition status
- teeth involvement and type of trauma (crown fractures, with or without pulp exposure, extrusive, intrusive or lateral dislocation and traumatic avulsion)
- occlusal post-traumatic alterations (open-bites, teeth with premature contacts)
- clinical examination of soft tissues to highlight alveolar bone exposure
- lesions involving oral mucosa, gums and tongue.

Recommendation 10

Careful clinical examination of the orofacial region must always be considered.

Recommendation strength: A.

Level of evidence: III.

Sub recommendation 10.1.

Any patient showing signs resulting from head/cervical trauma must be immediately sent to the Urgent Care Unit for proper evaluation.

Recommendation strength: A.

Level of evidence: IV.

Sub recommendation 10.2.

If signs suggesting mistreatment/abuse are present in a patient who has NOT been examined at the Urgent Care Unit, the dental practitioner is required to report it to the competent authorities, as this is a legally punishable crime (see Mistreatment and child abuse chapter).

Recommendation strength: A.

Level of evidence: IV.

Recommendation 11

Palpate bone profiles of the face and evaluate any alterations of mandibular movements (reduced/ asymmetrical mouth opening) which may suggest TMJ involvement (fractures, dislocations, endo-articular effusion).

Recommendation strength: A.

Level of evidence: III.

Recommendation 12

Vitality test response (often unreliable in young children), percussion test and mobility of the involved teeth must always be considered (see “Guideline on Management of acute dental trauma of American Academy of Pediatric Dentistry Revised 2011”).

Recommendation strength: A.

Level of evidence: I.

Recommendation 13

Dentinal/pulp exposure must be evaluated in case of spontaneous or stimulated local pain.

Recommendation strength: A.

Level of evidence: I.

### Radiological diagnostics

A radiographic examination must be performed in every dental trauma. Intraoral radiography is useful to evidence root fracture, tooth displacement or avulsion. If lip wounds are present, radiological examination of soft tissues may be necessary to identify the presence of foreign bodies.

In case of fractures, orthopantomography, teleradiography in antero-posterior projection and/or advanced imaging methods must be considered. Inverse Towne’s projection is recommended in very young and/or low-compliant patients thanks to the low radiation dose delivered.

Recommendation 14

Periapical intraoral radiography is recommended in case of traumatized permanent teeth, traumatic avulsion of deciduous teeth or intrusive dislocation.

Recommendation strength: A.

Level of evidence: VI.

Sub recommendation 14.1.

In case of crown infraction of a permanent tooth, with no dental mobility, intraoral radiography can be postponed; tooth vitality and percussion test response should be monitored every 6 months.

Recommendation strength: B.

Level of evidence: VI.

## Certificate of the dental trauma

Query: What criteria should be adopted by a dentist private practitioner to fill in a certificate in cases of dental or TMJ trauma occurring in growing subjects?

Keywords

Traumatic dental injuryDental traumaPediatric facial traumaMedico-legal issuesMedical certificateMedico-legal reportTMJ injury

Keywords have been searched alone and in association

Since the medico-legal reporting is regulated by national laws, The bibliographical search considered also national literature not included in the data banks described in the premise. A thorough evaluation of the selected study, no other studies emerged which were considered relevant for updating this chapter. Therefore the references’ list considered here is the same as that of the previous edition of the document

Introduction

The certificate is an attestation released by a health professional upon the request of the entitled person for different reasons or interests, reporting facts or evidence found during clinical activity [74–77].

In case of orofacial trauma, the dentist must provide appropriate diagnostic and therapeutic assistance to patients, but could be also requested to fill in a certificate.

This certificate is a deontological duty for doctors and dentists [78] and for all other health professionals. The certificate must be appropriately filled in in terms of content and using the correct formal features as a report, otherwise some relevant hypothesis of penal, civil or deontological wrongdoing can be raised especially against dentists who practice as public officials (dentists employed in the National Health Service, e.g.)

Very few and simple rules are dictated for writing a certificate (see. Additional file 1), that is hard to be standardized in a sort of pre set up form, since the content of the certificate varies according to the different traumatic lesions and the scopes (that in any case should be legal and licit) that the patient or the entitled person (parents of a child, e.g.) intend to pursue [75–82]:

The filling in of the certificate should follow a few simple rules and avoids a “standard printed form “approach as the nature of dental or TMJ trauma can vary greatly and involves possible.

compensation claims by individuals and their parents.

The certificate requested for supporting a claim for compensation, should:

- Be filled in at the same time as the clinical examination is provided
- Be clear and accurate in describing the trauma circumstances as reported by the patients, the clinical evidence, the diagnostic/therapeutic activity possibly performed and the prognosis. The certificate should detail if the patient need further exams or specialist referral (maxillo-facial surgeon, e.g.) for completing the diagnosis, specific treatments (provided by the dentist or other health professionals, such as a physiotherapist for TMJ trauma, e.g.), regular follow up with a possible re-evaluation of the diagnosis, prognosis and the related treatments.
- Specify if a radiological or complementary examinations (pictures, dental casts, etc) should be integrated in the diagnostic activity
- Highlight if the reported evaluations (for future treatments and costs, e.g.) should be submitted to a medico-legal assessment [85]

For dental traumas occurring at school or during road accidents, the onus-of-the proof for the lesion entity and nature is on the injured person, consequently the dentist must appropriately document the traumatic lesions, before the therapeutic interventions (the extractions of a fractured tooth, e.g.) will render the lesion not verifiable by a subsequent assessor. This preliminary registration of clinical conditions must always be performed, apart from emergency cases in which the treatment cannot be safely delayed (tooth replantation, e.g.). [86].

Recommendation 1

The certificate released as an attestation of the nature and the entity of traumatic lesions must accurately report the anatomical site of the trauma, the diagnosis and the prognosis.

Recommendation strength: A.

Level of evidence: V.

### Recommendation 2

The traumatic lesion must be documented (patient file, X-rays, pictures, etc.) in an appropriate way by the dentist in order to provide objective clinical evidence that is useful from a medico-legal and judicial point of view for both penal or compensation purposes.

Recommendation strength: A.

Level of evidence: IV.

## Dental trauma as a result of neglect and or abuse of children

Query: In the case of dental trauma, what are the elements that should lead clinicians to suspect non-accidental trauma?

Keywords

Dental injuriesDental traumaPediatric facial traumaChild neglectChild abuseDomestic violenceChild mistreatment

The keywords were searched in association with each other.

Inclusion criteria: literature research also included national bibliographic sources, not reviewed by databases, in order to find national data on child abuse. We considered all studies that answered the above question for a total of 51 works. Five works, which included relevant results, were filed and added to the previous bibliography.

Introduction

In recent years there has been an increase in reports of minors in conditions of hardship and/or complaints against adults who are perpetrators of violence [83].

In some European countries and in the USA, there are specific surveillance registers, from which it is possible to obtain data on this phenomenon. It has been reported that about 3–6 children in 1000 suffer abuse of various types.

In Italy, the real extent of child abuse or neglect remains an underestimated problem [83] due to the lack of a national register and therefore, it is not possible to quantify the phenomenon with precise data. The prevalence rate estimated by the PES 2000 National Group is about 8 per thousand [84].

The World Health Organization underlines that “Child abuse or maltreatment constitutes all forms of physical and/or emotional ill-treatment, sexual abuse, neglect or negligent treatment or commercial or other exploitation, resulting in actual or potential harm to the child’s health, survival, development or dignity in the context of a relationship of responsibility, trust or power [85].

In particular, there are four types of child maltreatment: physical abuse, sexual abuse, emotional abuse and neglect. Physical abuse occurs when the parents or guardians of the child practise or allow him/her to carry out acts, which may cause physical injury. The consequences of physical abuse are: injuries without lesions, skin and ocular lesions, visceral lesions, fractures, burns, multiple and repeated trauma [86].

Victims of child abuse can be found in all age groups, but the groups most at risk are new-born babies, infants and preschool children, particularly boys. The perpetrators are parents or caretakers in 90% of the cases, especially in young children [87].

Importantly, up to 50% of all physical injury associated with child abuse occurs in the head and neck region [88, 89]. A major study, performed on over 3385 subjects in developmental age with cranio-maxillo facial trauma, has shown that about 3.9% of cases was due to violence [90].

Regarding the trauma of the TMJ, some authors have shown that about 18% of the cases result from violence [91].

Fractures resulting from violence are most commonly associated with the angle region while those related to road traffic accidents usually involve the condyle, body and parasymphyseal fractures [92].

Regarding the face and neck, the oral cavity is less frequently affected by violence, but lesions at this level (bruising of the hard palate and springs, fractures tearing, burns of mucosae, etc.) can be easily detected by the health worker during an examination [93, 94].

Some authorities believe that the oral cavity may be a central focus for physical abuse because of its significance in communication and nutrition [95].

Generally, individuals subjected to abuse have lower levels of oral health [96, 97]. Therefore, the role of the dental team in identifying possible signs of abuse is clear, because during an examination it is possible to see beyond the inside of the oral cavity, as well as checking the head, the face and the neck of traumatized patients being assessed after a traumatic event.

In general, characteristics of abuse injury are polymorphism, multiple locations and colours due to the repetition of the abuse in different ways and at different times [98, 99]. A cohort study from Scotland reported that 59% of physically abused children had orofacial signs, such as bruises and abrasions that would be easily visible to a dentist. An apparent discrepancy in the trauma history provided by the parents and the injuries found on examination, or a delay in presentation along with a different history by each parent, should arouse suspicion in the clinician’s mind regarding non-accidental injury [48].

Although the oral cavity is a frequent site of sexual abuse in children, visible oral injuries or infections are rare. When oral-genital contact is suspected (in particular oral and perioral gonorrhea, or petechiae of the palate, particularly at the junction of the hard and soft palate) referral to specialized clinical settings equipped to conduct comprehensive examinations is recommended [95].

The presence of multiple lesions, dating back to different periods, in addition to specific injury (injuries caused by grabbing, fingernails, bites, cigarette burns etc.) should induce clinicians to take appropriate action in relation to the assistance profile (according to specific and appropriate specialized pathways) and in a timely manner and report the information to the relevant judicial authority. This is important not only to initiate judicial enquiries but also to put into place immediate procedures for the adoption of protection measures for the minor.

The evidence from the scientific literature revealed a discrepancy between the number of professionals who notice signs of violence against children and the number of professionals who report to the competent authorities. A study indicates that only 8% out of 28% of the dentists who noticed signs of violence against children reported this to the authorities [6].

An important problem is that, as various studies have shown, a significant number of dentists did not know which authorities to turn to in case of violence against children. Some authors observed that the dentists would contact the social and psychological services (21%), lawyers (10%), the police (9%), as well as the District Attorney and the Children’s Hospital (4%) [6].

Health professionals, including dentists, are obliged by law to report suspicions of abuse when - in the exercise of their function - they suspect or are certain that a crime has been committed which can be officially prosecutable (art.331, 332 e 334 Code of Criminal Procedure, art. 361, 362 e 364 Criminal Code).

Finally, in Article 24 of the new Italian ELA (Essential Levels of Assistance) regarding “Social and health assistance to minors, to women, couples, families “, provision is made that, in the context of district, domiciliary and territorial assistance, the National Health Service guarantees to women, children, couples and families all services, including home care, medical, diagnostic and therapeutic assistance.

These services are necessary and appropriate in different areas of activities, which include: prevention, evaluation, assistance and psychological support for minors in situations of hardship, in state of neglect or victims of abuse as well as the psychological and social support to family units in conditions of hardship including evaluation and psychological support for couples and minors for family custody and adoption.

Recommendation 1

In the case of dental trauma, the health professional must always ask if such trauma may have been caused by abuse or violence and, in the event that it is suspected, must report it to the competent authorities.

Recommendation strength: A.

Level of evidence: IV.

Recommendation 2

In the presence of a dental injury, the clinicians must perform, in addition to a careful evaluation of the oral cavity, an examination of the head and neck.

Recommendation strength: A.

Level of evidence: IV.

Recommendation 3

It is important to carefully evaluate the trauma of the TMJ as sometimes it can be a result of violence or abuse.

Recommendation strength: A.

Level of evidence: IV.

Sub-recommendation

In particular, the intraoral examination must verify the presence of possible wounds to the lips, tongue, palate and frenula. Signs of previous dental traumas may be due to repeated trauma caused by violence. The extraoral examination must include the inspection of the scalp (verification of hair-free areas), of the auricles and of the neck (verification of the presence of wounds or bruises). Furthermore, it is important to evaluate the skin of the anatomical areas indicated above in order to verify the presence of abrasions, recent or old bruises and non-self-inflicted bite marks. The eyes should be inspected for the presence of periocular bruising and the nose for the detection of septal deviations or blood clots.

Recommendation strength: A.

Level of evidence: IV.

## Conclusions

This document provides updated evidence-based guidance to assist dentists, paediatricians, surgeons, teachers, school and sport staff, parents in the prevention and first aid of dental trauma in children and adolescents and to provide a careful assessment of the medico-legal implications. Twenty-eight recommendations were given, divided in four domains. Regarding the first one “Dental trauma prevention strategies and health education”, recommendations in home, school and sport environment are given. Particular importance is given to the organization of training courses for teachers, school employees and students with the aim to identify and reduce risks of dental trauma and to provide the correct first-aid. About “First aid in orofacial and dental trauma”, recommendations are given regarding the immediate treatment of crown fracture and tooth avulsion and the general assessment of patient’s condition after the trauma. Diagnostic procedures are also discussed. In the “Certificate of the dental trauma”, it was assessed that the certification is a deontological duty for physicians and dentists and it needs to include a clear and accurate description of the trauma circumstances, clinical evidence, the diagnostic/therapeutic activity performed and the possible long-term prognosis. Few and simple rules helping to write a certificate are provided. Finally, in the “Dental trauma as a result of neglect and or abuse of children” section, it is highlighted that health professionals, including dentists, are obliged by law to report suspicions of abuse when, in the exercise of their function, they suspect or are certain that a crime has been committed which can be officially prosecutable.

## Supplementary information

**Additional file 1:.** Certificate form for orofacial trauma in children (DOCX 21 kb)

## Data Availability

Not applicable.

## Abbreviations

AAPD: American Academy of Pediatric Dentistry
ANDI: (*Associazione Nazionale Dentisti Italiani*) Italian National Association of Dentists
AP-TRX: Postero-anterior cephalograms
ELA: Essential Levels of Assistance
GTO: (*Gruppo tecnico sull’odontoiatria*) “Technical Group for Dentistry”
IADT: International Association of Dental Traumatology
LL-TRX: Lateral cephalograms
OPG: Panoramic X-ray
PNLG: *(Programma Nazionale per le Linee Guida)* Italian National Guideline Programme
SIMEUP: (*Società Italiana di Medicina di Emergenza ed Urgenza Pediatrica*) Italian Society of Emergency Paediatricians
SITD: *(Società Italiana di Traumatologia Dentale)* Italian Dental Traumatology Society
TMJ: Temporomandibular joint
WHO: World Health Organization
