# Supplementary material for: Italian guidelines for the prevention and management of dental trauma in children
Source: Ital J Pediatr. 2019 Dec 4;45:157. doi: 10.1186/s13052-019-0734-7 (PMC6894327; doi:10.1186/s13052-019-0734-7)
Supplement: Supplementary file 1 — Additional file 1:. Certificate form for orofacial trauma in children (DOCX 21 kb) [file 13052_2019_734_MOESM1_ESM.docx]

**Appendix 1**

**Certificate form for orofacial trauma in children.**

Date of the visit^i^ …………………………….first name……. Family name/surname………………….date of birth

……………………, accompanied by …………………….. (name of relative/tutor/teacher/other)^ii^, who reports ^iii^ that the child………..……. on (date of the trauma) ^iv^ ……………..approximately at (time of the trauma)……………………, during^v^ ………………………………………… due to …………………………..……………………….....had the trauma(specify)…………………………………………………………………………………………………then………

…….………………………………………………………………………………………………………………………………………

During the interview the subject complains of the following symptoms ^vi^ ……………………………………………………………………………..……………………………………………………………………………………………………………………………………………..……………………………………………………………………………………………………………………………………………………………………….

The clinical findings are ^vii^

……………………………………………………………………………………………………………..

………………………………………..…………………………………………………………………………………………………

……………………………………………………………………………………………………………………………………………

The diagnostic tests ^viii^ revealed ……………………………………………………………………………

…………………………………………………..………………………………………………………………………………………

Treatments performed ^ix^ ……………………….…………………………………………………………………………….

…………………………………..………………………………………………..…………………………………………………….…

Briefly the subject is deemed to be affected by ^x^ …………………………………………………………………….

…………………………………………………………….………………………………………………………………………………

The subject is discharged with the following prescriptions and recommendations ^xi^……………………….…………………………………………………………………………………………………………………

With a prognosis of ^xii^

…………………………………………………………………………………………………………………………………………

A follow up is necessary ……………………………….. for ^xiii^……………………………………………….……

……………………………………………………………………………………………………………………………………………

Dentist’s signature

date…………

**Notes. The following notes are explicative, but not exhaustive and are addressed to help the dentist, who is requested to write a certificate to attest the presence of traumatic injuries and to describe the diagnostic and therapeutic actions taken in each case, which can appropriately support a medico-legal pathway for a compensation claim.**

**^i^** Whenever the certificate is released on a date different from the date of the visit, do not alter or change the date of the certificate, but make it clear that the certificate and the visit happened on different dates. From a medico-legal point of view the dates have great relevance for understanding/attesting events.

**ii** It is important to specify who accompanies the child/adolescent and relates what happened. Moreover, it must be kept in mind that persons other than parents or legal tutors cannot give the consent for dental treatments. Only urgent treatment than cannot be safely delayed should be performed without consent.

Treatments that can be delayed without risk should be postponed to the moment when parents/tutors can give valid consent to treatment.

**iii** Terminology should make it clear that this part is based on what the accompanying person told the dentist and cannot be verified by the dentist himself.

**iv** The date and time at which the trauma happened should be reported, especially in relevant cases (tooth reimplantation, e.g.)

**v** In this section the dentist should report what the minor and the accompanying adult reported:

- the place where the trauma occurred and the cause. It should be kept in mind that different places (school, gym, home, etc) and causes (accidental falling, push, impact, aggression, etc) imply different compensation chances.
- the dynamic of the event (hit to the face, punch, etc.)
- the effect that the subject immediately suffered (loss of a tooth, gengival bleeding, limitations or pain at mouth opening or during chewing, breathing, speaking, etc.)
- subsequent events, for instance if the child was visited in a first aid facility or by different dentists or health professionals.

**vi** The symptoms complained by the minor should be described in this section (pain, difficulties in mouth opening, etc.)

**vii** The clinical examination must focus on the traumatic lesions of muco-gingival, osteo- dental and TMJ structures, etc. In cases of TMJ injuries the reduction of mouth opening should be reported with other clinical signs and symptoms.

**viii** This section is dedicated to describing what emerged from instrumental exams (radiographies, vitality tests, etc).

It is to be kept in mind that the occurrence of a traumatic injury must be demonstrated by the injured person, thus whenever the existence or the severity of the lesion could be put in doubt, one or more picture of tooth/injured area can greatly facilitate the subject in gaining compensation.

**ix** In this section the treatments carried out should be described in details (reimplantation, dental splint, restorations, etc). The medico-legal assessment of the compensation amount will require the damaged subject to provide the receipts for the afforded health care expenses due to the traumatic injuries.

**x** It is useful to provide a synthetic description of the case from a diagnostic, prognostic and therapeutic point of view. For instance: traumatic fracture and displacement of tooth n. 11, treated with tooth repositioning, splint and composite restoration.

**xi** Here the dentist can specify possible recommendations for a referral to a first aid facility or to the pediatrician or to a specialist (maxillo-facia surgeon, e.g.), or home-based recommendations (food, mouth hygiene,etc) or drug prescription or further instrumental exams, etc.

**xii** In this section the practitioner should describe the prognosis:

- the need for patient to be referred to A and E/ hospital
- The prognosis and the needed follow up. Mention if the prognosis needs to be reviewed after subsequent follow ups (in case of pulpar shock, e.g.) or after a suitable therapy (drugs or physiotherapy for TMJ injuries, e.g.)

The prognosis is crucial from medico-legal point of view both for the practitioner (see the duty of reporting some specific crimes) and for the injured subject (being possibly eligible for compensation)

It must be remembered that, according to Italian criminal code, the crime “personal lesions” must be reported by health professionals to the Public Prosecutor (professionals are not requested to be sure about the occurrence of the crime; the mere possibility is enough for health professional to be obliged to report) in the following cases:

*-voluntary* (aggression, abuse, domestic violence, etc) when the illness duration is > 20 days or a permanent impairment of stomatognathic functions occurred (tooth loss, e.g.)

Should the practitioner suspect abuse, an appropriate and attentive approach must be used in the best interest of the minor. Dentist could turn for a consultation to the child’s pediatrician and a attentive observation of typical abuse lesions (bite marks, burn wounds, cuts to the scalp, bruises of the face, etc) that generally are multifocal, given the reiterated abuse and which show different stages of healing

The criminal code provides no punishment for an undue report, but an abuse of a minor is a serious crime possibly involving one parent/relative, thus dental practitioners are requested to be proceed carefully. Nevertheless, dentists must not disregard or undervalue the possible occurrence of abuse that affects with similar frequency all areas/ social classes/ religious faiths.

The occurrence of lesions possibly linked to abuse due to bullying or sexual aggression must be appropriately considered in adolescents.

-*culpable* (lesions due to negligent behaviour) due to violations of laws on the workers protection for injuries and occupational diseases and that caused a duration of the illness > 40 days or a permanent impairment of stomatognathic functions (a tooth loss, e.g.). These cases are quite rare as very few young people work. The Law 41/2016 established that also in case of serious and very serious personal lesions caused by traffic accidents (art. 590 bis penal code) the health professionals are obliged to report to Public Prosecutor, even though a possible modification of the law is currently being studied

For injury compensation, the prognosis is extremely relevant, first to understand when the lesion has stabilised and a reliable assessment of the injuries can be provided (about the vitality of the tooth, e.g.) and then the temporary (disturbances, pain, alterations that temporarily affected the subject, but then stabilised in a permanent impairment or disappeared) and the permanent impairment (permanent reduction of validity of one or more functions of the subject) can be assessed and evaluated.

**xiii** This clarification is very relevant for cases for which the tooth/tissue/structure should be re-evaluated after appropriate examinations (pulp shock, e.g.) up to the moment the final prognosis can be reliably formulated and eventually the case can be submitted to medico-legal evaluation, thus avoiding concrete risks of over-under evaluation of lesions/impairments (both for criminal and civil cases). When the minor’s parents request a certificate as exclusive support for a compensation claim the dentist should be sure that a proper medico-legal assessment of the damage has been provided, otherwise the case should be referred to a medico-legal assessor. The referral should be mentioned in the certificate.
